# Deaths from Inhalation of Chloroform

**Published:** 1854-01

**Authors:** 


### *Deaths from the Inhalation of Chloroform.*

[The London Lancet of October 29th, contains the report of two cases of death from the inhalation of Chloroform—one at the University College Hospital, October 6th, and the other at St. Bartholomew's Hospital, October 21st. The letter we copy entire, with the post-mortem examination, as every particular in a case of this kind is full of interest. We also insert the series of rules, by a distinguished surgeon of Paris, which are appended in the Lancet to the report.—*Ed. Bost. Med. and Surg. Jour.*

The patient was a girl of loose habits, 22 years of age, who had been in this hospital two years before the present admission. She was then laboring under an affection which was long looked upon as syphilitic; there was, in fact, considerable discharge from the vulva, and within the vagina was seen an ulcer which was thought to be of a specific nature; but it turned out to be a cancroid growth, situated just at the entrance of the vagina. It had, on former occasions, been observed that no secondary symptoms were occurring, though the sore presented a certain amount of induration; there was no pain, but the discharge was pretty considerable, and harassed the patient much.

Mr. Paget, having resolved to destroy the tumor, gave the preference to the actual cautery, and hoped that by this means he should succeed in freeing the patient from the inconvenience she was suffering. A fortnight before the day when the inhalations of chloroform had a fatal issue, the ulcerated surface was touched for the first time, when the patient had also inhaled chloroform. She had been thrown into an incomplete state of anæthism, for she started when the heated iron came in contact with the sore; she was therefore made to inhale more chloroform, and fell into perfect narcotism, from which she subsequently recovered very well.

On the 21st of October, it was thought advisable to repeat the operation, and the girl was brought into the operating theatre. Dr. Black, warden to the College, who administers chloroform by appointment, placed upon the patient's mouth the ordinary tin and leather inhaler, which covers nose and mouth, and which is always used in this Hospital. When she had been placed on the table, Dr. Black applied the apparatus, and she continued to inhale the anæsthetic agent very quietly for about ten minutes before it took any effect on her. All at once the patient was noticed to present an unusually dusky countenance; the pulse became weak and fluttering, and the breathing irregular. Mr. Paget had not as yet begun to operate, and the whole attention was now turned to the state of the girl, and every effort used to recall her to life. Artificial respiration was first employed in the manner advised by Ricord, the air being thrown into the lungs from mouth to mouth. As this, however, did not succeed, an opening was made between the thyroid and cricoid cartilages, and artificial respiration continued by means of a tube passed into the aperture, to which a pair of bellows were adapted. In order to rouse the system, brandy and water was thrown up the rectum. Whilst these measures were energetically carried out, a warm bath was being prepared, and the patient was placed into it as soon as it was ready, artificial respiration being persevered in while she was immersed. During the continuance of these efforts, Dr. Burrows and Dr. Black detected now and then a pulsation at the wrist; but all these endeavors proving useless, galvanism was had recourse to. The shocks produced very strong spasms but no efforts at breathing; and it was plain that the only measure that could be relied upon was artificial respiration. This was continued with the greatest perseverance, but to no avail, and it soon became apparent that all the efforts at reviving the poor girl were perfectly useless. The whole amount of chloroform which had been inhaled was below two drachms, and, as stated above, the apparatus was the usual one, viz: the leather and tin case for nose and mouth, with the upper aperture and sponge for pouring in the chloroform.

*Post-mortem Examination made twenty-four hours after Death, conducted by Mr. Paget.*—There was general congestion of the brain, but not very marked, the only veins much congested being those at the posterior part, the blood being in a very liquid state. The puncta were not larger than usual, and the blood, which had been placed in the jar, did not coagulate. The ventricles contained an ordinary amount of fluid, and the pons Varolii presented normal features on a section being made through it. The only peculiarity worth noticing, (and the same had been observed in the patient who died from the effects of chloroform some time ago, under the surgical care of Mr. Loyd,) is that the blood was found liquid in the veins, and remained so after it had been put aside. The kidneys were somewhat congested; the left one was found scarred from previous disease, when the proper tunic was drawn off, and it was supposed that this was the cause of disease in early life. The peritoneum was thickened on the surface of the liver, and the left kidney was full of liquid blood. The spleen was adherent to the diaphragm from previous general peritonitis. The stomach was full of undigested food, and still the patient had stated that she had had no dinner; it is supposed that she took bread from her locker, and had potatoes given her by her fellow patients. On the mucous membrane of the stomach some coagulated milk was adherent, but the viscus itself was quite healthy, as was also the pancreas, of which there was a small offset attached to the serous surface of the jejunum. The heart was altogether flabby, but decidedly *not fatty*; the right ventricle was of the ordinary size, and slightly mottled at the upper part, the muscular tissue being rather of a thin texture, and generally pale. The lining membrane of the ventricle was rather thickened, and the paleness of the heart formed rather a contrast with the florid tint of the voluntary muscles, but the viscus did not present the character of fatty degeneration.

Now what do we learn from these accidental deaths, and the account of the post-mortem examinations? 1st. That the fatal effects may ensue in a very short or comparative long time, (three minutes in one case, and ten in the other.) 2d. That a fatty heart will cause death to occur in a much shorter time than is necessary when this organ is sound. 3d. That a perfectly healthy heart is no preservative from the fatal effects of chloroform. 4th. That a previous complete anæthesia by chloroform is no guarantee that a subsequent one will be harmless. 5th. That even the artificial respiration from mouth to mouth, which has been much extolled, may fail at a certain advanced period of the anæsthesia. 6th. That patients may fall victims to chloroform though in an excellent state of general health. 7th. That intemperance seems a counter-indication to the use of chloroform. 8th, and lastly. That accidents of the kind described above will happen with the best and most practised hands.

The next question is—Whether we can offer any suggestion as to the means of avoiding the sad results which we have just mentioned? On this point we gladly refer our readers to the excellent papers which from time to time have been published on the subject, and shall just extract from M. Bauden's memoir such advice as may be considered of value under the present circumstances:

1. Never go, intentionally, beyond the limit of cutaneous insensibility.

2. The management of chloroform may be divided into three stages—before, during, and after the inhalations.

3. *Before: Counter-Indications.*—Study the patient's constitution; find out whether there exist organic lesions of the heart or lungs: these would be a counter-indication, as are also asthma, aneurism, phthisis, chlorosis, anæmia, chorea, &c., and predisposition to cerebral congestion.

4. The patient's mind should be perfectly calm, and the medical attendant should speak of chloroform as a boon, when carefully administered.

5. The patient should be wishing for anæsthsia, and have full confidence in his medical adviser. If he should feel any apprehension or gloomy forebodings, chloroform should be steadfastly refused.

6. Patients have in all times died from the fear or pain of operations; but the influence of *fear* is now no longer taken into account, and chloroform accused of all the mischief.

7. Chloroform must never be given but for operations of a certain importance, and patients should be fasting.

8. Attention should be paid to the debility which naturally follows serious operations and considerable loss of blood, for the organism thus loses its power of resisting the influence of anæsthetic agents.

9. The operating room should be of good dimensions, easy of ventilation, and every article necessary in case of danger should be at hand.

10. *During the Inhalation.*—Chloroform should be administered in hospitals by persons specially appointed for the purpose, and in town by practitioners who make it their exclusive occupation.

11. The quantity of chloroform should be carefully measured, about fifteen minims being taken at once.

12. The length of time during which the patient is inhaling should be counted upon the watch, as also the pulse and the number of respirations. Note should be taken of the force and frequency of the pulsations of the heart; if the latter fall *below sixty*, the inhalation should be stopped.

13. The patient should be in the recumbent position, the head slightly raised by a pillow; and should be given doses of fifteen minims, the time between them being made gradually shorter.

14. The handkerchief should be first held at a little distance, and gradually brought nearer the face, the patient being spoken to in a kind and encouraging manner.

15. The latter should be frequently asked, while he is being pinched, what is done to him; and when he begins to answer with ill-humor, when you pinch him, he is on the point of losing the faculty of sensation.

16. As soon as he answers no more, feeling is abolished; the handkerchief should be taken away and the operation begun, for we should never wait until muscular relaxation is complete.

17. Excitement, which often marks the first degree, is a mark that the handkerchief should be removed, far from being kept on as is generally practiced.

18. The time has now come to watch the heart and the respiration. On the slightest retardation, and if the symptoms of anæsthesia go on or are even increased, means should be immediately taken to bring back the insensibility to the first degree.

19. When spasms of the larynx or much cough occur; if foam come to the mouth; if the pulse falls; if breathing becomes embarrassed; if there appears any mark of syncope or cerebral congestion, the inhalations should at once cease.

20. Slight struggling may be resisted, but violent excitement, and the exclamation of "I am choking," should be followed by the immediate removal of the handkerchief.

21. For long operations the inhalations should be intermitted, and the chloroform may be resumed as soon as the patient begins to sigh or move about. Anæsthesia has in this manner has been kept up for one hour.

As to the means to be used in cases of threatened death, M. Baudens enumerates most of those which were used in the two cases which we have adduced above.

